# Closed-Loop Attention Restoration Theory for Virtual Reality-Based Attentional Engagement Enhancement

**DOI:** 10.3390/s20082208

**Published:** 2020-04-14

**Authors:** Gang Li, Shihong Zhou, Zhen Kong, Mengyuan Guo

**Affiliations:** Department of Micro/nano Electronics, Shanghai Jiao Tong University, Shanghai 200240, China; zsh87387887@sjtu.edu.cn (S.Z.); kz1143356997@sjtu.edu.cn (Z.K.); guomengyuan618@sjtu.edu.cn (M.G.)

**Keywords:** attention restoration theory, virtual reality, EEG, attention, engagement

## Abstract

Today, as media and technology multitasking becomes pervasive, the majority of young people face a challenge regarding their attentional engagement (that is, how well their attention can be maintained). While various approaches to improve attentional engagement exist, it is difficult to produce an effect in younger people, due to the inadequate attraction of these approaches themselves. Here, we show that a single 30-min engagement with an attention restoration theory (ART)-inspired closed-loop software program (Virtual ART) delivered on a consumer-friendly virtual reality head-mounted display (VR-HMD) could lead to improvements in both general attention level and the depth of engagement in young university students. These improvements were associated with positive changes in both behavioral (response time and response time variability) and key electroencephalography (EEG)-based neural metrics (frontal midline theta inter-trial coherence and parietal event-related potential P3b). All the results were based on the comparison of the standard Virtual ART tasks (control group, *n* = 15) and closed-loop Virtual ART tasks (treatment group, *n* = 15). This study provides the first case of EEG evidence of a VR-HMD-based closed-loop ART intervention generating enhanced attentional engagement.

## 1. Introduction

Today, as media and technology multitasking becomes pervasive, the majority of young people face a challenge regarding their attentional engagement (that is, how well their attention can be maintained) [1]. A study of min-by-min observations showed that the typical university student could not focus on their work for more than 3 to 5 min even during a short 15-min class session, because their minds were still thinking about what might be occurring in virtual worlds, coaxing them to get back to the smartphone, tablet, or laptop to “check in” [2]. Such constant attempts to multitask not only create challenges to the development of their cognitive control functions [3,4,5], but also have a negative impact on their real-world activities, including bad sleep [6], poorer school/workplace performance [7,8], and an increased level of stress and anxiety [9,10]. Therefore, there exists a need for new methods to enhance attentional engagement. A recent study showed that six weeks (20–30 min per week) of training with a closed-loop digital meditation software delivered on a smartphone/tablet improved attentional engagement in young adults [11]. Here, “closed-loop” refers to a novel meditation training approach where the training difficulty can be adjusted in a rapid manner (e.g., every dozen seconds), according to the participant’s performance. Actually, apart from meditation, attempts to boost attentional engagement in a drug-free manner also involved physical exercise, cognitive training, video games, brain stimulation, and exposure to nature [12]. All of these approaches are building on the foundation that our brain modifies itself—a phenomenon known as neuroplasticity in the context of cognitive neuroscience [12,13]. However, unlike those active interventions (e.g., physical exercise, cognitive training, video games, brain stimulation) that involve hard work of the participant, exposure to nature is an approach that is quite the opposite.

Exposure to nature is a kind of passive intervention. Walking and running in real nature, or passive viewing of natural scenes through images and videos are the widely seen forms in this approach [13]. Essentially, there are no demanding attentional tasks involved. Since the 1980s, accumulating evidence shows us that nature surroundings have restorative or stress-reducing effects [14,15,16,17]. Specifically, Kaplan et al. who developed attention restoration theory (ART) suggested that natural environments have properties that attract involuntary attention and, thus, allow a depleted directed attention capacity to recover so that cognitive fatigue can be reduced [18]. Here, the term “natural environments” must consist of the following four key components [19,20,21]: (1) “being away”, which refers to the sense of being psychologically detached from present worries and demands; (2) “soft fascination”, which involves the fascinating objects that capture one’s attention in a bottom-up fashion and generate minimal top-down responses; (3) “extent”, which refers to the degree that people feel immersive and engaging; (4) “compatibility”, which refers to our intrinsic motivation to stay in certain environment.

In a more recent review article about ART, Garside et al. showed some empirical evidence of attention-based benefits, such as improved working memory in both healthy people and those with psychological conditions, which were generated from “before and after” behavioral measurements [13]. However, in this review article, there were also some studies with mixed findings using different outcome measures of attention, as well as one that did not offer support for ART with outperformed control groups instead of the intervention groups [22]. It is likely that differences in the quality of the four aforementioned components themselves contribute to different outcomes [23]. For example, the usage of artificial settings, i.e., urban parks and gardens [24,25], apparently degraded the sense of “being away” and “soft fascination” if compared to real natural scene. Moreover, the awareness of “extent” makes it hard to believe that people would feel immersive and engaging by just viewing scenery images and videos. Furthermore, the recruited healthy population probably would not have a strong and “compatible” motivation if compared to the people who are suffering from cognitive fatigue. More importantly, none of them (all ART-related studies in Reference [13]) reported neural evidence. Thus, attentional engagement, a state that describes how well the attention-based benefits can be maintained, remains unclear.

Unlike previous ART studies that adopted ready-to-use natural resources, our approach involved designing, developing, and testing an ART-inspired virtual reality (VR) software program that integrates key restorative components of traditional ART with a real-time electroencephalography (EEG)-based closed-loop algorithm to monitor their vigilance levels—an indicator of hard mental work over prolonged periods of time [26]. When engaging with this program, users were firstly instructed to close their eyes for 3 min to get their EEG baseline parameter, and then they began “exposure to nature” while their vigilance levels were simultaneously monitored using EEG. They were not given explicit instructions on the best strategy via which to achieve a low vigilance level, but they did know that their EEG was being recorded and that it was being used to change the natural surroundings as a reward-based feedback for their vigilance levels (for example, fog disappears when low vigilance level detected). We define this closed-loop approach as CL-ART or “extent”-enhanced ART, which allows the interaction between human and natural environment in a personalized manner and offers regular feedback on the effectiveness of relaxing the mind from top-down demands through engagement in a strong bottom-up driven activity. In the context of cognitive neuroscience, attention is one of the three cognitive control abilities (the other two are working memory and goal management) [12]. Thus, the term “top-down” here refers to internal guidance of attention based on prior knowledge, willful plans, and current goals, while “bottom-up” refers to attentional guidance purely by externally driven factors to stimuli that are salient because of their inherent properties relative to the background [27]. Given that the core idea of ART is exposure to nature, ART-induced attention refers to bottom-up attention.

VR is a communication medium that leads an individual to perceive experiences as if they were physically present in that environment [28]. Such VR experiences not only provide easier access to difficult-to-arrange real-world situations, but also allow brain activity to be recorded in a controlled environment [29,30]. Therefore, VR environments are increasingly being used by researchers to simulate social interactions and natural events [31]. This trend is especially clear since the cost of VR was reduced from tens of thousands of United States (US) dollars a decade ago to the current hundreds of dollars with the development of consumer-friendly VR head-mounted display (HMD) technology in 2016 [32].

VR environments are designed using three technologies: non-immersive VR [33], semi-immersive VR [34], and immersive VR (IVR) [35]. There are two commonly used forms of IVR [35]: cave automatic virtual environments (CAVEs) and HMDs. A CAVE is a specially designed room in which the walls, ceiling, and/or floor are covered with a screen that can project virtual images or videos. An HMD is a VR headset that positions two small screens in front of both eyes, completely blocking out the physical world including the user’s body. All current state-of-the-art IVRs adopt consumer-friendly HMDs, including personal computer (PC)-powered HMDs (i.e., HTC Vive™), smartphone-based HMDs (i.e., Samsung Gear™), and all-in-one HMDs (i.e., Oculus Quest™), in order to achieve immersive effects in a manner that is both simple and inexpensive. More importantly, given that external factors may distract attention [36], VR-HMD has the inherent advantage of effectively limiting influences of external distraction on attention if compared to a conventional two-dimensional (2D) platform [37], highlighting the necessity of using VR-HMD in the current study.

The goal of this study was to assess both behavioral performance and EEG-based neural metrics on a perceptual discrimination task that was executed before and after two types of Virtual ART interventions: standard ART (ST-ART) and CL-ART. The two kinds of ART interventions were developed by our laboratory and delivered using a consumer-friendly VR-HMD platform, to quantitatively evaluate the impact of the “extent” component on attentional engagement with a high degree of ecological validity and experimental control. Given the higher degree of engagement that CL-ART is expected to offer (compared to ST-ART), we hypothesized that participants engaged in the CL-ART tasks would generate better post-intervention behavioral performance, as well as neural correlates associated with heightened attentional engagement.

## 2. Materials and Methods

### 2.1. Virtual ART Tasks

**Figure A1:**
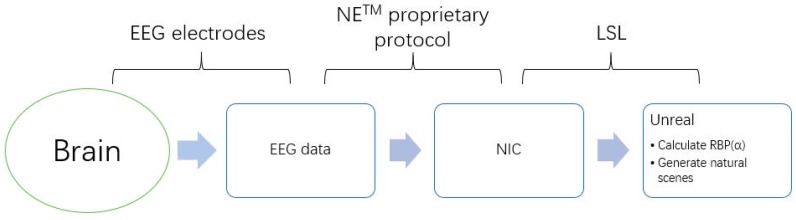
The data flowchart of the combined VR–EEG system.

**Figure A2:**
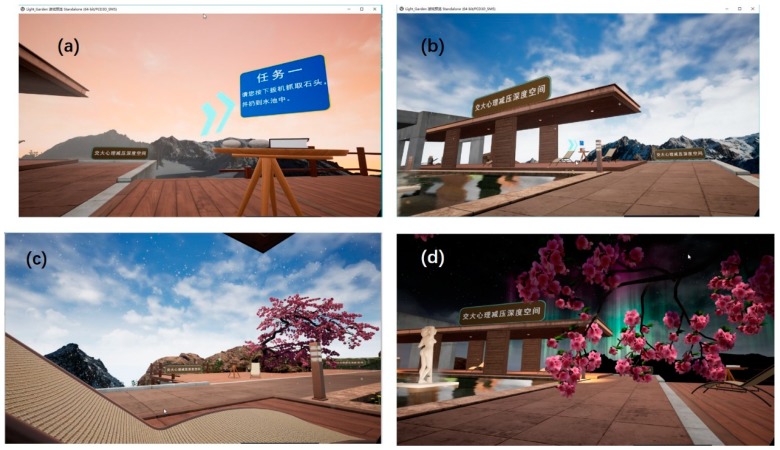
Screenshots from ST-ART showing (**a**) the task 1, (**b**) the reward following task 1 (clear sky), (**c**) the light snow during task 2, and (**d**) the reward following task 3 (aurora).

Virtual ART is a VR-HMD software developed at Shanghai Jiao Tong University (SJTU)’s bio-circuits and system laboratory to assess the effectiveness of ART with a high degree of ecological validity and experimental control. There were two types of virtual tasks in this study: ST-ART (control group) and CL-ART. Both were developed from the principles of Kaplan’s theory and delivered in HTC Vive™—a flagship consumer-friendly VR-HMD platform powered by an NVIDIA GeForce GTX 1070 graphic card. As can be seen in Table 1, to have a fair comparison of both kinds of tasks, all set-ups were in their best form. For example, head and limb movements were allowed in ST-ART tasks to achieve broader field of view and naturalistic interaction in the context of VR, while such movements were not allowed in CL-ART tasks to avoid the negative impact of serious motion artefacts on EEG. In the meanwhile, except for “extent”, we carefully kept other components identical. The duration of both tasks was the same 30 min. More technical details can be found in Figure A1. A few screenshots of ST-ART are presented in Figure A2. Additionally, a helpful video demo link for CL-ART can be found in Appendix A as well.

**Figure 1:**
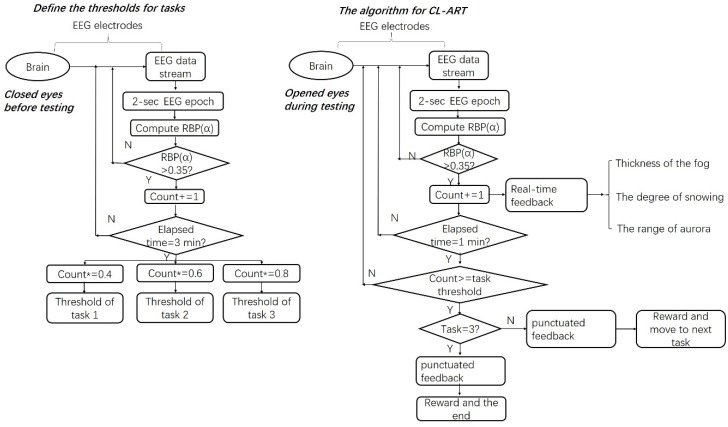
The thresholds for each task (**left**) during CL-ART experiment and the algorithm for CL-ART (**right**).

For CL-ART, EEG data were collected through an eight-channel wireless EEG recording device (StarStim 8™, Neuroelectrics Inc, Barcelona, Spain), which uses a high-resolution, high-speed analog-to-digital converter (24 bit at 500 sampling rate), and supports Bluetooth connection. The conventional wet electrodes were used and placed at O1, Oz, and O2 regions, which are proven locations that are highly correlated with the user’s vigilance level in the context of a real-time and real-world scenario [38,39]. The ground and reference electrodes were connected together and placed on the right earlobe using an ear clip. An external electrode was placed below the lower eyelid to record eye movements. The collected EEG data were firstly filtered using a 4–30-Hz bandpass filter and averaged across O1, Oz, and O2 channels. Then, two types of EEG-based feedback (as shown in Figure 1) were provided to participants: (1) real-time feedback, which was based on the values of relative α band power (RBP(α), see Equation (A1) in Appendix A) calculated every 2 s, indicating the dynamics of within-task vigilance level, and (2) punctuated feedback, which was based on the total counts of the values of 2-s RBP(α) being larger than 0.35 (35%) every 1 min, indicating the vigilance level between tasks. The specific thresholds between tasks were weighted values of the highest counts of RBP(α) > 0.35, which were determined using min-by-min observations during the initial 3-min EEG baseline testing session. The weights were 0.4, 0.6, and 0.8 for tasks 1, 2 and 3 respectively. Here, the values of weights and the value of 0.35 were based on feedback from our pilot testing, in which the principle of selecting these values was that the participants should not consider it too easy or too difficult. The lab streaming layer protocol [40] was used to synchronize the vigilance level and the Unreal Engine-based virtual natural scenes.

### 2.2. Outcome Measures

As shown in Table 2, behavioral performance and EEG-based neural metrics captured during a perceptual discrimination task (visual oddball) that was executed before and after the two ART interventions were utilized as our outcome measures to assess the potential benefits of attention. Specifically, the commonly used response time (RT) [41,42,43] and event-related potential (ERP: P3b latency) [44] were used to evaluate the general benefits of attention; then, the response time across trials (RTVar [11,45]) and inter-trial coherence at the frontal midline theta band (ITC(θ) [11,46]) were used to assess the attentional engagement. Finally, the long-range frontal-posterior inter-electrode coherence at the theta band (IEC(θ)) was used to investigate the brain functional connectivity [42].

#### 2.2.1. Visual Oddball

**Figure A3:**
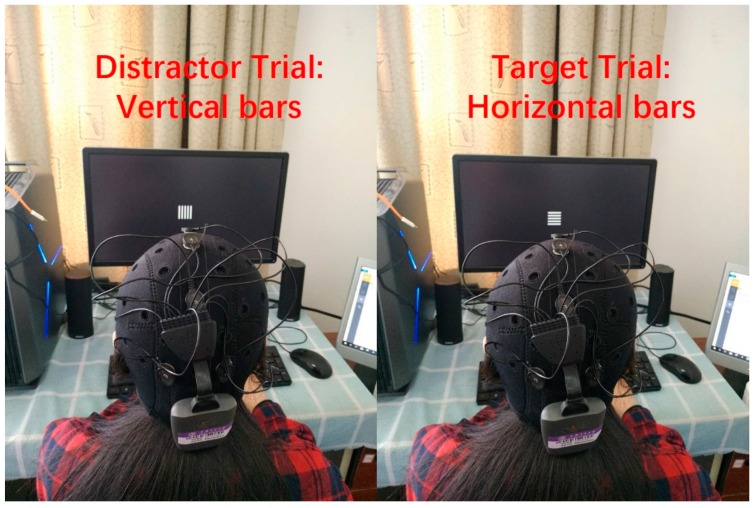
Visual oddball tasks.

For oddball testing, we used the same EEG cap (i.e., StarStim 8™). During the experiment, participants were instructed to keep their eyes fixated on a central fixation cross on the computer screen and keep their dominant-hand middle fingers on a home position on the keyboard until the appearance of a given stimulus type (target/distractor). Upon the appearance of these stimuli, participants were instructed to press the “enter” key using their middle fingers if it was a target (horizontal zebra stripes on the center of the screen; see Figure A3 in Appendix A) and to not release their middle fingers from the home position if it was a distractor (vertical zebra stripes on the center of the screen). In this kind of perceptual discrimination paradigm, the participant’s voluntary attention is directed to a rarely presented target stimulus while their EEG response to the target stimulus is simultaneously recorded; therefore, the neural evidence of the restored voluntary attention can be investigated. Each oddball task contained 300 trials and a ratio of 1/4 of target/distractor stimulus. The two trial types were presented randomly with no more than four consecutive trial types of either kind in a row. The inter-trial interval of 1500 ± 500 ms and a stimulus duration of 100 ms were used. Thus, the total time for each oddball task was approximately 10 min.

#### 2.2.2. EEG Data Pre-Processing

A low-pass filter with a cutoff frequency of 30 Hz and a high-pass filter with a cutoff frequency of 0.5 Hz were applied to remove power line noise and the direct current drift, respectively. The filtered EEG signals were then corrected using the mean of each channel (including Ext, Fpz, Fz, Cz, Pz, O1, Oz, and O2), and decomposed into eight independent brain sources by independent component analysis (ICA). The prominent artefactual components, such as eye blinks, eye movements, and muscle activity were removed by using ADJUST version 1.1 (an automatic algorithm for ICA-based EEG artefact removal [47]). Next, the target epochs of −1000 ms to +1000 ms were created and further cleaned of excessive peak-to-peak deflections, amplifier clippings, and other artefacts, using a voltage threshold of 100 μV. All neural metrics were calculated using custom Matlab™ scripts and EEGLab v14.1.2. (an open-source Matlab plugin developed by Swartz Center for Computational Neuroscience, La Jolla, CA, USA; http://www.sccn.ucsd.edu/eeglab).

#### 2.2.3. P3b

ERP time-locked to target trials were generated from the pre-processed EEG data recorded from participants while they performed the oddball task. All ERPs were baseline-corrected using a −200 to 0 ms time period, with the window of interest interrogated being 250–600 ms post-stimulus for P3b—an ERP component which is hypothesized to reflect the allocation of attention resources [44], and which was shown highly correlated with motor or cognitive action, such as pressing a button [48]. Given our focus on RT-based metrics for behavioral data, we focused on P3b latency in the Pz channel, which is the location where the P3b is commonly reported to reach its maximum amplitude [49].

#### 2.2.4. ITC(θ) and Long-Range IEC(θ)

ITC is a measure that reflects the extent to which synchronization occurs from trial to trial in EEG at a particular frequency [46]. The frontal midline ITC in the theta frequency band, ITC(θ), is a widely used measure of electrophysiological response consistency [11,50]. Thus, we used it as our neural metric of the degree of attentional engagement. Here, the theta frequency band (4–7 Hz) at the Fz channel and the phase-locking value (PLV_ITC_) were used as the input and output, respectively, in ITC(θ) analysis. The values of PLV_ITC_ ranged anywhere between 0 and 1. A larger value denotes perfect phase-locking synchronized across trials and, thus, a higher degree of attentional engagement.

IEC is a commonly used measure of phase consistency across electrodes [51]. Here, we calculated the PLV of the theta frequency for Fz–Pz coherence (PLV_IEC_) as our neural metric of brain frontal-posterior functional connectivity. Similarly, the values of PLV_IEC_ ranged anywhere between 0 and 1. A larger value denotes perfect phase-locking synchronized across the Fz–Pz area and, thus, stronger brain frontal-posterior connectivity. All ITC(θ) and IEC(θ) values were baseline-corrected using a −200 to 0 ms time period, with the window of interest interrogated being 0–700 ms post-stimulus, as average RTs occur <700 ms.

### 2.3. Participants, Procedure, and Statistical Analysis

A total of 50 interested students of SJTU signed up this study through online advertisements (where we named the designed virtual environment as “*the deep space of SJTU for mind relaxation*”). All of them were screened online for moderate to high trait anxiety (a score greater than 40 on the trait-anxiety subscale of the State-Trait Anxiety Inventory questionnaire, STAI [52]—a standard clinical measure of trait and state anxiety). With two time points (“before and after” the ART interventions), two groups (ST-ART/CL-ART), five measurements (RT, RTVar, P3b latency, ITC(θ), and IEC(θ)), and assuming a moderate repeated measures correlation (*r* = 0.5) and 0.05 α level, we calculated that *n* = 30 would yield 93% power to detect a change with a medium effect size (0.5). This effect size is not uncommon, as shown in Reference [11].

Thus, a reasonable sample size (*n* = 30, 21.3 ± 1.58 years of age, 13 females) who met the trait anxiety criterion (STAI score: 48.1 ± 6.05) were further invited to schedule a lab session. All participants had normal or corrected-to-normal vision, had no history of stroke, traumatic brain injury, or psychiatric illness, and were not taking psychotropic medication. We only enrolled participants who did not have a history of VR experience to balance the impact of familiarity of user interface on task performance and neural metrics. All participants were paid $15/h for their participation and gave written informed consent before participation.

The recruited 30 students were randomized to either the ST-ART group (control group, *n* = 15) or the CL-ART group (treatment group, *n* = 15) using a random number generator (seed =123, https://stattrek.com/statistics/random-number-generator.aspx). Prior to virtual ART tasks, students completed the consent document and state-anxiety subscale of STAI. After that, they were equipped with an EEG cap to practice for 2 min before doing the real visual oddball task. Immediately after the oddball task, they put on the VR headset and were instructed to close their eyes for 3 min (i.e., sham/real baseline test for ST-/CL-ART tasks). Then, the software guided them in a 5-min practice session before experiencing either 30-min ST-ART or CL-ART tasks. After the ART tasks, they were instructed to take a 10-min break while the VR headset was taken off. Immediately after the break time, they did the oddball task again.

All behavioral and neural data were analyzed using standard one-way repeated ANOVA with ART task types (ST/CL) as the between-subject factor, where behavioral and neural data refer to the difference before and after the same ART type. We used the difference (diff) of “before–after” comparison if there was an expected decreased trend or “after–before” comparison if there was an expected increased trend (as shown in Equations (1) and (2)) to keep showing a positive difference. Paired *t*-tests were used to further compare the impact of the within-subject factor (before/after the intervention) on participant performance and neural response. All statistical analyses were done using SPSS 19.0 with an α level of 0.05.

(1) $$\mathrm{Metrics}\_\mathrm{Diff}=Metrics_{before}-Metrics_{after},\;where\;Metrics\in \;\left\{RT,\;RTVar,\;P3b\;latency\right\}$$

(2) $$\mathrm{Metrics}\_\mathrm{Diff}=Metrics_{after}-Metrics_{before},\;where\;Metrics\in \left\{ITC\left(\theta \right),\;IEC\left(\theta \right)\right\}$$

### 2.4. Study Design

The study was designed in a double-blinded manner. Double-blinding began at the point of recruitment, when all participants were informed that they were being recruited for a study designed to test the efficacy of software interventions for mind relaxation. Thus, participants in both the CL-ART group and the ST-ART group thought that they were part of an active treatment group. One study coordinator (S.H.Z.) was in charge of the group assignments. M.Y.G. who collected data was blind to group assignment. All data analysis was done by Z.K. and G.L.

## 3. Results

First of all, the results of anxiety level were analyzed, in order to investigate the consistency of “compatibility” element between ST-ART and CL-ART group. Then, as shown in Table 2, the attention levels associated with behavioral metric, RT, and neural metric, P3b latency, are presented. Next, we analyzed the attentional engagement using the behavioral metric, RTVar, and neural metric, ITC(θ). It is important to note that the correlates of behavioral and neural metrics are presented. Finally, IEC(θ) was analyzed to explore the brain functional connectivity between the two groups before and after the 30-min VR experience.

### 3.1. Anxiety Level

**Figure 2:**
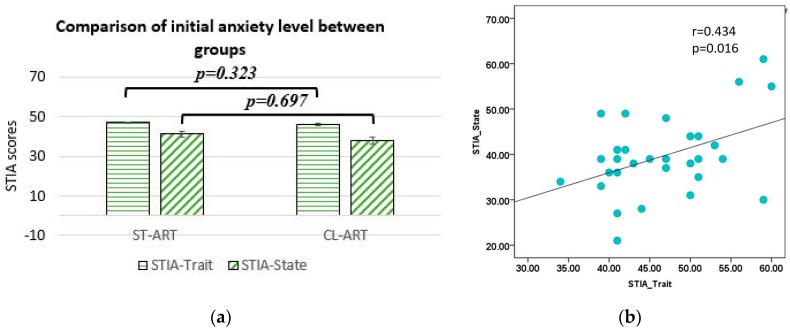
(**a**) One-way ANOVA analysis of state and trait anxiety State-Trait Anxiety Inventory questionnaire (STAI) scores between ST-ART and CL-ART groups; (**b**) scatter plots for state and trait anxiety STAI scores.

To have a fair comparison, first of all, we analyzed the trait and state anxiety STAI scores between the ST-ART and CL-ART group. As shown in Figure 2a, there was no significant difference in both types of scores between the two groups (trait anxiety scores: F(1, 29) = 0.155, *p* = 0.697, mean (M) = 46.067 ± 1.714 for ST-ART and M = 47.067 ± 1.873 for CL-ART; state anxiety scores: F(1, 29) = 1.012, *p* = 0.323, M = 38 ± 2.434 for ST-ART and M = 41.2 ± 2.048 for CL-ART), indicating the same initial anxiety level or motivation (see “compatibility” element in Table 1) for each group. Moreover, we found that the on-site state anxiety scores highly correlated with trait anxiety scores (*p* = 0.016, *r* = 0.434, see Figure 2b), indicating the good consistency of the earlier sign-up session and later invited lab session.

### 3.2. Attention Level

**Figure 3:**
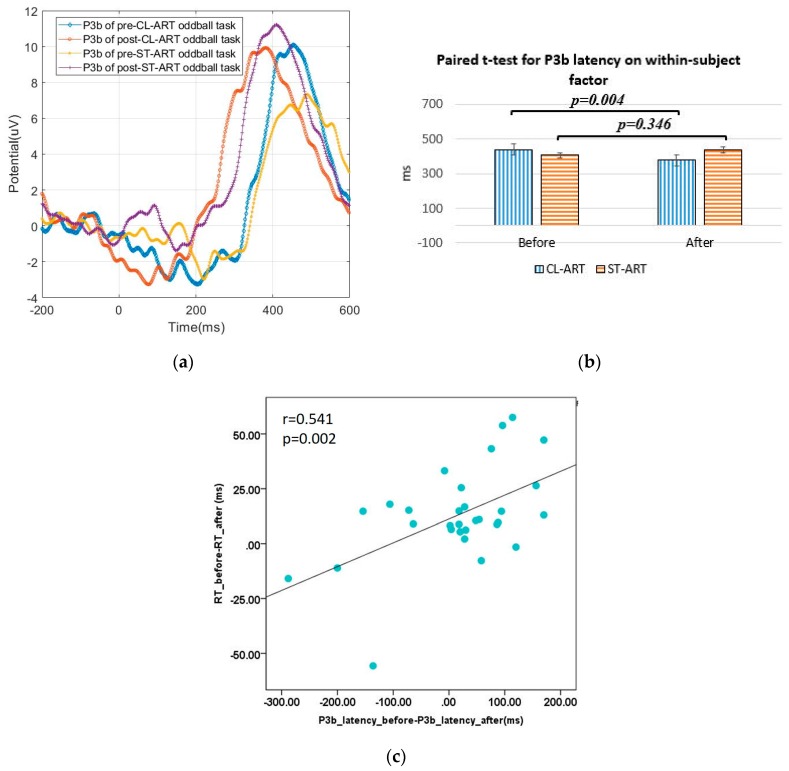
(**a**) The group grand average for P3b captured before and after the two kinds of ART interventions; (**b**) paired *t*-test and subject means for P3b; (**c**) scatter plots for RT difference and P3b difference.

For RT, we found a significant difference in P3b latency (F(1, 29) = 6.581, *p* = 0.016) between groups. The paired *t*-test of P3b latency before and after CL-ART intervention (t(14) = 3.481, *p* = 0.004) further shows that participants responded, on average, 62 ms faster in the post-CL-ART oddball task (M = 377.733 ± 17.116 ms) than in the pre-CL-ART condition (M = 439.867 ± 8.964 ms), indicating the neural evidence of enhanced attention level. This trend is clearly shown in Figure 3a and b by comparing the grand average of P3b between ST-ART and CL-ART groups. Furthermore, as shown in Figure 3c, we found a significant correlation between RT difference and P3b difference (*p* = 0.002, *r* = 0.541), indicating the highly correlated behavioral and neural evidence.

### 3.3. Attentional Engagement

**Figure 4:**
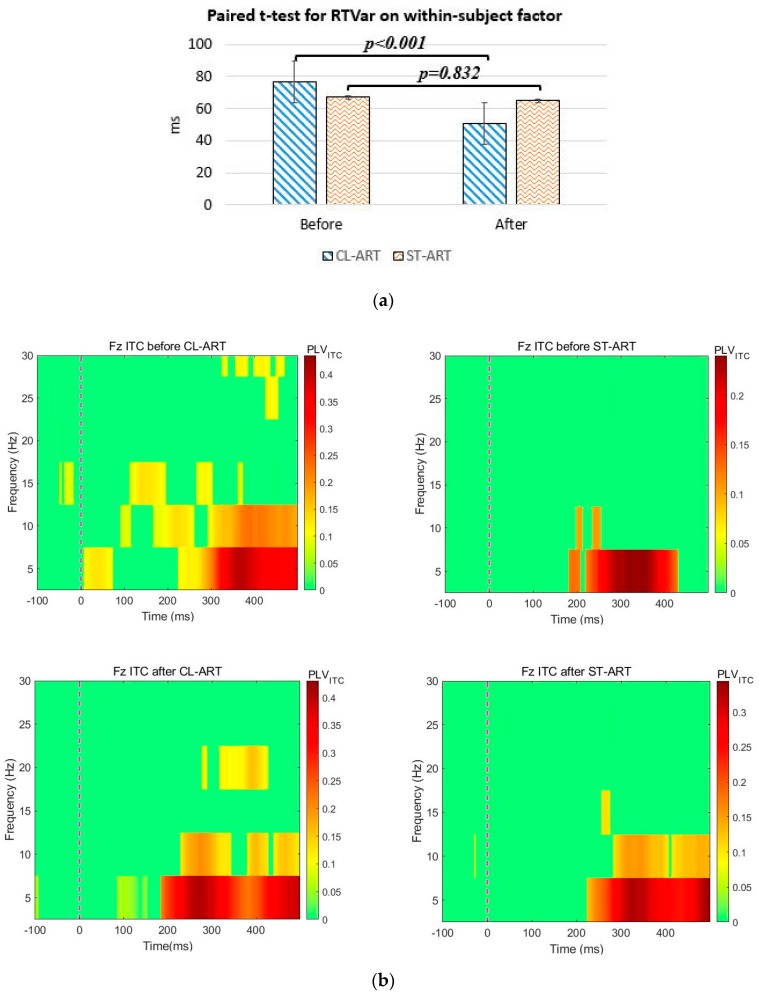

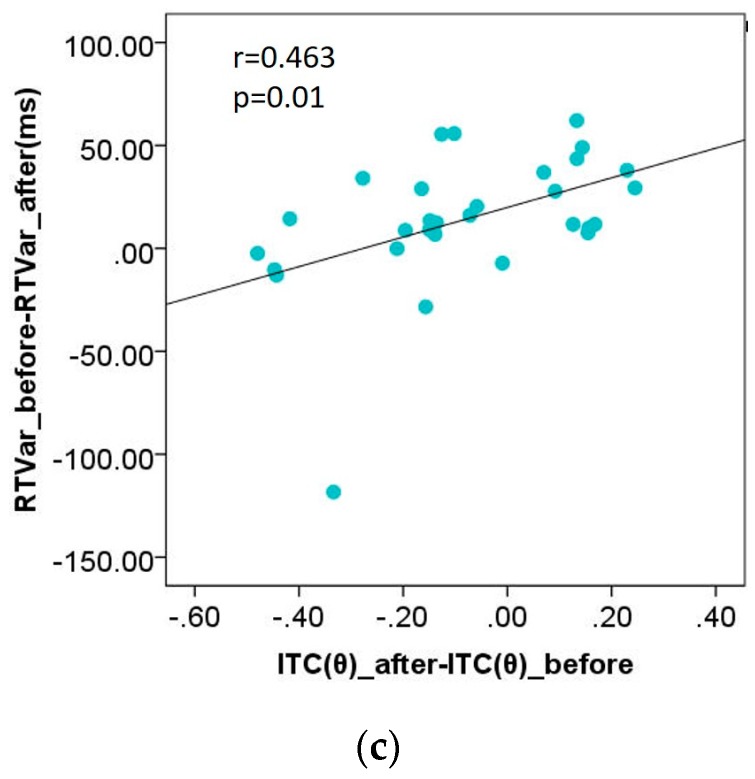
(**a**) Paired *t*-test and subject means for RTVar; (**b**) the group grand average for ITC captured before and after the two kinds of ART interventions; as can be seen here, the highest phase-locking value (PLV; deep red) in the “after CL-ART” condition occurred in the bin of 200–300 ms, which was clearly faster than that in the bin of 300–400 ms in other conditions; (**c**) scatter plots for RTVar difference and ITC difference.

We found a significant difference in RTVar data between groups (F(1, 29) = 4.256, *p* = 0.048), with M = 2.278 ± 10.532 ms for ST-ART and M = 25.932 ± 4.533 ms for CL-ART. The paired *t*-test (*t*(14) = 5.721, *p* < 0.001) further reveals that participants responded with less variability in RT in the post-CL-ART oddball task (M = 50.929 ± 3.940 ms) if compared to the pre-CL-ART condition (M = 76.862 ± 4.335 ms), as shown in Figure 4a. Moreover, we observed that there was a most prominent difference in averaged values at 300–400 ms post-stimulus ITC(θ) between groups (F(1, 29) = 14.215, *p* = 0.001). Such a group difference in ITC(θ), as shown in Figure 4b, was found by comparing the 100-ms bins one by one in our time window of interest (see Table 3). These behavioral and neural findings indicate that CL-ART may be indeed superior to ST-ART for improving attentional engagement. The highly correlated RTVar difference and ITC(θ) difference (*p* = 0.01, *r* = 0.463; see Figure 4c) further reveals the robustness of these findings.

### 3.4. Brain Functional Connectivity (IEC(θ))

Similar to ITC(θ), we investigated each 100-ms bin in our time window of interest (see Table 4); however, no significant difference in IEC(θ) was observed, indicating that there was no prominent improvement in frontal-posterior brain functional connectivity following the two kinds of ART experience.

## 4. Discussion

### 4.1. Comparison to Prior Work

Previously, the enhancement of general attention level was observed using traditional ART intervention and behavior-based metrics within a reported duration of as short as 15 min [53] or as long as weeks or years [54,55]. The upgrade from traditional ART intervention to this closed-loop approach aims to study EEG-based neural metrics and further verify the feasibility of improving attentional engagement by enhancing one of the restorative components, “extent”.

The present findings reveal that the attentional engagement is improved in young adults when a single 30-min ART-inspired closed-loop intervention is used as opposed to a standard ART intervention, evidenced via both behavioral and EEG-based neural metrics. This more engaging closed-loop approach was actually accompanied by improved sensitivity and greater fidelity in assessing cognitive abilities [56]. This was especially important when these abilities were often assessed with validated pencil-and-paper approaches or, now more commonly with these same paradigms deployed on either desktop or laptop computers, in a format that regularly reveals low test sensitivity in children [56,57]. In addition, the neural evidence-based attentional benefits occurring in such a short time period were observed in another study [58], in which the author reported that feedback training of α rhythms resulted in fMRI-proved changes in brain networks after a single 30-min session (i.e., the exactly same time period of intervention with current study). However, the difference between the current study and Reference [58] is that (1) we used ART-based natural scenes as a feedback paradigm rather than effort-demanding racing game, and (2) we used EEG to investigate the trial-to-trial neural metrics in attentional engagement instead of fMRI-based changes in brain structure or network. Of course, these approaches are not mutually exclusive; they are complementary, and participants will likely achieve the most beneficial outcomes if they use them concurrently. For example, the brain functional connectivity measured by IEC(θ) could perhaps be improved by using more effort-demanding tasks instead of ART-like natural scenes.

### 4.2. Significance for Future Study

The present findings demonstrate that the closed-loop personalized Virtual ART intervention achieved a higher degree of engagement compared to conventional Virtual ART intervention, highlighting the importance of real-time neurofeedback between human and virtual environment. This exploratory study is especially pertinent given that Jyoti et al. recently proposed the next generation of cognitive training approach that incorporates rapid, real-time, performance-driven, adaptive task challenges and performance feedback [59]. More importantly, with the development of miniatured bio-sensors and wireless VR-HMD, a unique and novel commercial VR platform [60], which combines EEG recording and eye-tracking technologies, emerged to facilitate Jyoti’s idea. Although this study was conducted in healthy adults only, related work demonstrated that populations with physical and intellectual disabilities can benefit from VR in capturing and maintaining attention [61,62]. Thus, the findings presented here have practical implications for assessing attention-related diseases, including sensory processing dysfunction [63], attention deficit hyperactivity disorder [64], and mild impaired cognition [65] in the context of clinical research. Given that previous work [63,64,65] showed the feasibility of using 2D-based attention assessments and interventions for these diseases, we surmise that the use of a more engaging and enjoyable VR-HMD may indeed follow a similar or even better results.

### 4.3. Limitations and Conclusion

While the improvements on attention level and attentional engagement were observed during a 10-min perceptual discrimination task (visual oddball) carried out 10 min after the CL-ART intervention, no conclusions can be made regarding the long-lasting effects of the observed attentional benefits. Moreover, without accompanying MRI neuroimaging to assess the neural mechanisms of the observed intervention effects, it is not possible to make strong conclusions as to the neural underpinnings of the CL-ART effects. Therefore, follow-up studies driven by the positive findings reported here are planned with (1) larger and more diverse populations, (2) advanced neuroimaging technologies, and (3) a greater variety of perceptual discrimination tasks.

To our knowledge, this is the first study to quantitatively evaluate the impact of the “extent” component on user attentional engagement after ST-ART and CL-ART interventions that were delivered on a consumer-friendly PC-powered VR-HMD platform with both behavioral and EEG measurements. The experimental results support the conclusion that the CL-ART tasks may indeed be superior to ST-ART tasks in terms of user engagement. This is especially pertinent given that a total of 14.35 million VR-HMD units were sold in 2017 to 2019 worldwide [66], highlighting the oncoming wave of such accessible technology for researchers and clinicians to utilize these tools in ways never before achieved. Furthermore, if one takes advantage of the burgeoning all-in-one VR-HMD platform (e.g., Oculus Quest), the benefits of this closed-loop ART approach can be extended to larger numbers and more diverse populations.

## Figures and Tables

**Table 1:** Comparison of standard attention restoration theory (ST-ART) and closed-loop attention restoration theory (CL-ART) tasks.

Restorative Components		Virtual ART Tasks	
		ST-ART	CL-ART
**Being away**		Secluded home surrounded by snowy mountain, meadows, and pool.	
**Soft Fascination**		Morning (clouds and fog), afternoon (snowing), and night (cricket sound and aurora), as well as a shared scene from morning to night: motion of cherry blossom in the breeze.	
**Compatibility**		Anxious university students who voluntarily participated in this study.	
**Extent**	**Task 1 (for morning scene)**	The thickness of fog is randomized. VR controller is used to capture stones on the table and throw them into the pool one by one.	The thickness of fog is controlled by EEG at 2-s interval. Wireless Xbox 360 joystick is used to walk around in any accessible virtual space while relaxing mind to make fog vanish.
	**Reward**	Bright sun makes fog vanish and task 2 starts.	The degree of snow is controlled by EEG at 2-sec interval.
	**Task 2 (for afternoon scene)**	VR controller is used to turn on the music player on the table, listening to classic music while walking around (by teleport function) in any accessible virtual space until the end of music.	Wireless Xbox 360 joystick is used to walk around in any accessible virtual space while relaxing mind to turn on the music player on the table, listening to classic music until the end of music.
	**Reward**	Once the music is played, the default light snow converts into heavy snow. By the end of music, heavy snow stops and task 3 starts.	The snow stops and task 3 starts.
	**Task 3 (for night scene)**	No specific task, just walking around (by teleport function) in any accessible virtual space.	Wireless Xbox 360 joystick is used to walk around in any accessible virtual space while relaxing mind to call aurora.
	Reward	Aurora appears. The range of the aurora is randomized, updating every 2 s.	Aurora appears. The range of the aurora is controlled by EEG at 2-s interval.

**Table 2:** Summary of outcome measures. RT—response time; ITC—inter-trial coherence; IEC—inter-electrode coherence.

Task	Types of Measure		Implications
	Behavioral	Neural	
**Visual oddball**	RT	P3b latency	General attention level
	RTVar	ITC(θ)	Attentional engagement
	/	IEC(θ)	Brain functional connectivity

**Table 3:** Summary of one-way ANOVA analysis results for ITC(θ) difference.

Bins (ms)	*p*-Value	PLV (Mean ± Standard Error)	
		CL-ART	ST-ART
0–100	0.002	0.042 ± 0.167	0.027 ± 0.011
100–200	0.199	−0.035 ± 0.032	0.026 ± 0.034
200–300	0.031	−0.102 ± 0.058	0.075 ± 0.052
300–400	0.001	0.041 ± 0.042	−0.202 ± 0.493
400–500	0.008	0.032 ± 0.043	−0.175 ± 0.058
500–600	0.678	−0.021 ± 0.052	−0.051 ± 0.050
600–700	0.898	−0.050 ± 0.048	−0.042 ± 0.038

**Table 4:** Summary of one-way ANOVA analysis results for IEC(θ) difference.

Bins (ms)	*p*-Value	PLV (Mean ± Standard Error)	
		CL-ART	ST-ART
0–100	0.179	0.034 ± 0.026	−0.027 ± 0.035
100–200	0.149	0.068 ± 0.032	−0.021 ± 0.051
200–300	0.487	0.047 ± 0.031	−0.006 ± 0.067
300–400	0.467	−0.004 ± 0.043	0.054 ± 0.066
400–500	0.797	0.036 ± 0.043	0.055 ± 0.058
500–600	0.841	0.053 ± 0.053	0.070 ± 0.050
600–700	0.724	0.014 ± 0.045	0.035 ± 0.038

## Appendix A

This appendix contains some technical details about the Virtual ART, supplementary figures from Virtual ART and oddball tasks, and a link for the demo video of CL-ART.

1. Technical Details about Virtual ART

The contact quality of EEG electrodes and scalp was color-coded in a software, called NIC (NE^TM^), which pairs with the EEG recording device (black: no signal; red: very poor signal; orange: fair signal; green: good signal). The collected EEG data were continuously displayed and stored on a PC by NIC. In the meanwhile, the C++ version of the lab streaming layer (LSL) protocol was used to transfer the EEG data from NIC to the Unreal game engine, and then the RBP(α) was calculated to control the natural scenes (see Figure A1 and Equation (A1)), where, Power is the fast Fourier transform (FFT) power, *z_i_* = {θ, α, β}.

(A1) $$RBP(\alpha )=\frac{Power(\alpha )}{\sum_{i=1}^{3}Power(Z_{i})}\times 100\%$$

2. Supplementary Figures for Virtual ART

3. Video Demo for CL-ART

Available online: https://www.youtube.com/watch?v=2vSbK_QS_kE

4. Supplementary Figures for Visual Oddball Tasks
